# Medical Students’ Knowledge and Adherence to Paediatric Choking Rescue Manoeuvre Guidelines: A Multicentre Study of Medical Education Curricula

**DOI:** 10.3390/healthcare13121441

**Published:** 2025-06-16

**Authors:** Jakub R. Bieliński, Riley Huntley, Dariusz Timler, Klaudiusz Nadolny, Filip Jaskiewicz

**Affiliations:** 1 Emergency Medicine and Disaster Medicine Department, Medical University of Lodz, 90-419 Lodz, Poland; jakub.bielinski@umed.lodz.pl (J.R.B.); dariusz.timler@umed.lodz.pl (D.T.); 2 Faculty of Applied Science, School of Nursing, University of British Columbia, Vancouver, BC V6T 2B5, Canada; rileydj@student.ubc.ca; 3 Department of Emergency Medical Service, Faculty of Medicine, Silesian Academy in Katowice, 40-555 Katowice, Poland; prrm.knadolny@interia.pl; 4 Collegium Medicum, WSB University, 41-300 Dabrowa Gornicza, Poland

**Keywords:** first aid, paediatric, children, foreign body airway obstruction, choking, airway, back blows, chest thrusts, abdominal thrusts, Heimlich manoeuvre

## Abstract

**Background/Objectives:** Bystander first aid in paediatric choking is crucial. It ought to be universally comprehensible and backed up by evidence-based guidelines. However, there still are inconsistencies in guidelines worldwide. The objective of this research was to assess the knowledge of medical students on paediatric choking rescue manoeuvres and their educational backgrounds in order to evaluate the impact of differences in educational curricula. **Methods:** Medical students from a total of 12 universities across Canada, Libya, and Poland were surveyed online. The questionnaire assessed the respondents’ experience, training, and knowledge in first aid regarding foreign body airway obstruction in infants and children. **Results:** Out of 324 responses, 290 were evaluated. Although the students studied in only 3 countries, they represented 37 countries of origin. A total of 7 new reference groups were created based on guideline identification. A comparison of 4 clinical scenario questions revealed that certain training providers communicate recommendations more effectively to medical students, as their guidelines seem to have better knowledge retention. **Conclusions:** There are important differences in medical student knowledge, possibly due to discrepancies in training programs and guidelines. Variability was found in body position, anti-choking suction devices, blind finger sweeps, and medical follow-ups. More research is needed to standardize training and improve worldwide choking management outcomes.

## 1. Introduction

Foreign body airway obstruction (FBAO), commonly known as choking, is one of the leading causes of accidental paediatric death worldwide, with 75% of all FBAO cases occurring in children younger than 3 years old [1,2]. In developed countries, around 300 to 600 fatal accidents occur annually, but the situation is significantly more dire in underdeveloped countries, where fatalities occur at twice the rate [3,4,5]. Despite increasing recognition, there has been a steady rise in the frequency of paediatric FBAO over an extended period [5]. Additionally, recent research indicates that almost half of fatalities happen before the administration of any rescue interventions, indicating disregard in first aid recognition and management of choking or considerable delays in providing appropriate rescue manoeuvres [6,7].

Severe airway blockage may result in respiratory failure and cardiac arrest [8]. However, even in cases of partial blockage, life-threatening complications, such as airway swelling and inflammation, can arise from the delayed extraction of foreign objects [9]. Evidence suggests the high importance of early bystander intervention in reducing potential risks and averting fatal outcomes for paediatric FBAO incidents. Prompt, effective action by bystanders and medical personnel is critical in choking emergencies, with the potential to prevent deterioration and improve survival.

A key determinant in successfully assisting a choking child is the rescuer’s level of first aid knowledge and skills [10,11]. Providers need a clear understanding of guidelines to deliver effective care. Disturbingly, evidence indicates a lack of widespread competence among the general population in dealing with paediatric FBAO [12,13]. Even among caregivers of children, there is a notable lack of knowledge, with some studies suggesting that only 5% to 30% of parents and teachers demonstrate adequate awareness of the problem [14,15,16].

Individuals who received first aid training show significantly greater knowledge than those without, with evidence suggesting that around half of those trained have knowledge and comprehension levels above the minimal level [13]. These findings suggest that healthcare professionals with comprehensive first aid training should possess the best understanding of critical lifesaving techniques, such as airway management, during choking incidents.

However, there is insufficient high-quality evidence to conclusively determine the level of knowledge and skills among healthcare providers who may provide care to children. According to current evidence, self-evaluations of paediatric residents in foreign body removal show a low level of competency, and this does not appear to improve over time with increasing work experience [17]. Prehospital emergency medical care personnel trained in basic life support, who are often called to paediatric emergencies, also demonstrate inadequate awareness regarding first aid algorithms for choking children and knowledge of the steps involved in FBAO treatment for an unresponsive paediatric patient [18].

The limited number and low quality of available data create a research gap, which needs further scientific investigation to improve evidence of healthcare providers’ knowledge and competence in paediatric FBAO management. Medical students should receive training in first aid and possess knowledge on how to properly perform rescue manoeuvres for a choking child. Medical students may encounter choking cases in clinical or community settings. They may also need to educate parents, thereby equipping caregivers with the skills necessary to manage choking incidents. Basic prehospital emergency medicine skills are imperative for junior healthcare providers [19]. Although their competence in managing adult patients is generally extensive, there is limited information available concerning their proficiency in paediatric cases [20]. This raises the concern that adult-focused training may come at the expense of paediatric FBAO management.

Emergency management of choking is based on weak recommendations with very low certainty of scientific evidence [21]. Guidelines and algorithms regarding first aid for choking children differ across regions and organizations [22]. In infants (children aged 0–1 year) who have preserved consciousness but an ineffective cough, the guidelines are consistent throughout and recommend back blows as initial (first) and chest thrusts as secondary (second) rescue manoeuvres. Similarly, recommendations on when and how to implement cardiopulmonary resuscitation (CPR) are in accordance with each other. However, algorithms regarding first aid for choking children (age definitions varying by guideline, often ranging from 1 year to puberty) vary drastically (Table 1) [23,24,25,26,27,28,29,30].

These discrepancies may influence the effectiveness of FBAO management by both laypeople and healthcare providers. The lack of standardization could plausibly contribute to confusion among first aid providers, reducing their confidence and potentially affecting adherence to best practices. This, in turn, might result in variable applications of rescue manoeuvres and less predictable outcomes in time-sensitive emergencies. Further research is needed to assess the knowledge and competence of medical students in managing paediatric FBAO. This could improve future educational and training systems, help standardize divergent recommendations and guidelines, and ultimately, enhance outcomes for this vulnerable patient population.

With the aim of improving educational and training systems while simultaneously boosting the standardization of recommendations and guidelines and ultimately improving outcomes for vulnerable patient populations, future research should assess the knowledge and competence of medical students in managing paediatric FBAO. Gathered data can enrich scientific knowledge with previously unavailable information. Comparing distinct first aid education systems will reveal their advantages and disadvantages, enabling the identification of the most accessible and high-quality educational approach.

This study examines the knowledge and its sources of multinational medical students regarding first aid for paediatric FBAO. Given the absence of a universally validated and recommended emergency management algorithm based on scientific evidence, it is essential to assess medical students according to their specific, regional guidelines and training.

The detailed objectives of this study are as follows:

- Assess the preferred primary and secondary first aid rescue manoeuvres for children and infants with FBAO and determine their alignment with guidelines that influenced each participant’s training program.
- Assess awareness of optimal positioning of the infant or child for these techniques.
- Evaluate how medical students manage a paediatric patient who has lost consciousness due to FBAO.
- Examine respondents’ knowledge and attitudes regarding the use of anti-choking suction devices, the blind finger sweep manoeuvre, and the need for medical follow-ups for a child with restored airway patency.

## 2. Materials and Methods

### 2.1. Study Settings

A descriptive, cross-sectional study was conducted using an online survey developed by the authors. Data was collected from April 2024 to July 2024 from medical students in Canada, Libya, and Poland. The study was approved by the Bioethics Committee at the Medical University of Lodz (number RNN/156/24/KE). The reporting of this study follows the STROBE (Strengthening the Reporting of Observational Studies in Epidemiology) statement (Supplementary Material S1).

### 2.2. Data Source

Data was collected through an online survey questionnaire (Supplementary File S1) created using the Microsoft Forms software (ver. 16.89.1 (24091630), Microsoft Corporation, Redmond, WA, USA) and distributed to participants via a QR code or direct link. To control for duplications of the responses, the survey platform was configured to allow only one response per email address.

The survey was designed to grant participants the greatest possible freedom to choose their course of action in order to examine, in the statistical analysis, the alignment of answers to the specific guidelines and not the correctness nor the order of the chosen rescue manoeuvres, as there is no globally unified algorithm of first aid management.

The first part of the questionnaire consisted of 4 sociodemographic questions and 4 questions devised to gather information about respondents’ training process and experience. The second part of the questionnaire consisted of 4 open-ended questions: 2 focused on the first aid management of FBAO in an infant and 2 addressing FBAO in a 3-year-old child. After each question, medical students were asked to specify the positioning during the chosen lifesaving techniques. Respondents were also asked how they would manage a paediatric patient who has become unconscious due to FBAO. Further questions explored their views on the use of blind finger sweeps, the necessity of medical follow-ups after performing rescue manoeuvres, and their opinions on anti-choking suction devices.

Validation of the questionnaire was carried out using face and content validation techniques involving 24 participants from the target population. These respondents took part in structured interviews conducted by one of the researchers, during which they were asked to evaluate the questionnaire items for clarity, comprehensibility, consistency of interpretation, and neutrality. Adjustments were made based on feedback, using a five-point Likert-type evaluation scale.

This approach is consistent with established best practices for cognitive interviewing and pretesting, especially for open-ended and scenario-based instruments that are not intended for psychometric scaling. In such contexts, sample sizes ranging from 5 to 25 participants are considered sufficient for detecting major issues with question design and interpretation [31,32]. Therefore, the use of 24 pilot participants was deemed adequate for the aims of this stage of validation.

Full-scale psychometric validation (e.g., exploratory factor analysis or Cronbach’s alpha) was not applicable given the structure and purpose of the instrument.

### 2.3. Participants

Medical students attending accredited institutions in Canada and Libya, as well as international students enrolled in the English Division at the Medical University of Łódź in Poland, were invited to participate to improve comprehension of the survey’s language and cohesion within the studied groups. The choice of institution was dictated by previous cooperation, and the authors recognized the opportunity to conduct the study while maximizing multinational features to the extent that was available to them. Convenience sampling was used to recruit potential participants through recruitment posters displayed at educational facilities and emailed invitations to participate. Participation in the survey was voluntary and anonymous. An online information sheet explaining the purpose of the study and confidentiality of data was made available to all participants. Consent was implied by completing the survey. The inclusion criteria were willingness to participate in the study and a completed questionnaire. Although no a priori sample size calculation was performed due to the absence of a definable population frame, the adequacy of the sample was assessed through a post hoc sensitivity analysis. This study involved comparisons between 8 training council groups using categorical variables and chi-squared tests.

We chose 290 medical students to participate in the study through a convenience sampling method. However, the post hoc sensitivity analysis revealed that this sample size was sufficient to detect medium effect sizes (Cohen’s w = 0.25) with 89.7% power at α = 0.05 using chi-squared tests. This suggests that the collected sample was sufficiently large to identify statistically significant differences in the knowledge and adherence rates between groups.

### 2.4. Statistical Analysis

Statistical analysis was performed using the SPSS Statistics software (version 29.0.2.0), IBM Corp., Armonk, NY, USA) and RStudio version 4.3.3 (R Foundation for Statistical Computing, Vienna, Austria). Quantitative variables are presented using basic descriptive statistics: the arithmetic mean (x), standard deviation (SD), median (Me), minimum (Min), maximum (Max), interquartile range (IQR), and percentages (%). For comparisons where the expected frequencies were greater than five, distributions were analysed with the chi-squared test for independence. When the expectations were less than five, Fisher’s exact test was chosen. Where the initial test indicates significance (*p* < 0.05 was considered significant, and *p* < 0.01 was considered highly significant), either the Benjamini–Hochberg False Discovery Rate post hoc test or the Bonferroni correction post hoc test was chosen to control for type-I errors, dependent on the dimension of the contingency table. Open-ended responses were consecutively reviewed manually with a qualitative content analysis and categorized thematically, and recurring patterns were summarized descriptively. This process was independently conducted by three authors, and the findings were then jointly reviewed by the entire group to ensure standardized and consistent conclusions.

## 3. Results

**Figure 1:**
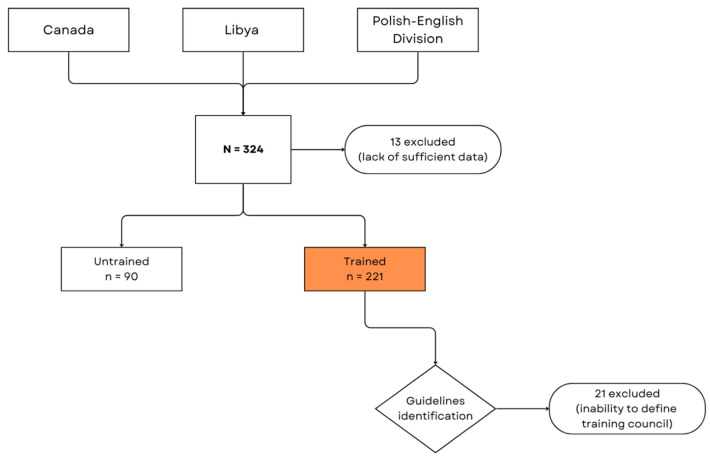
Flow chart of this paper methodology, process, and structure.

A total of 324 responses were received, and 13 responses were ineligible due to missing data and were discarded. Respondents were assigned to reference groups based on their responses to the question, “Have you received training on how to manage airway obstruction caused by foreign objects in children and infants?”. Respondents, who self-reported no training (*n* = 90) were assigned to the untrained group and served as the control. Trained respondents were assigned to the guideline identification group (*n* = 221) according to self-reported data of which the training council’s guidelines they had been taught and were subsequently categorized accordingly. Some respondents (*n* = 21) were excluded due to their inability to identify their training provider or with which guidelines/standards training was conducted (Figure 1).

**Figure 2:**
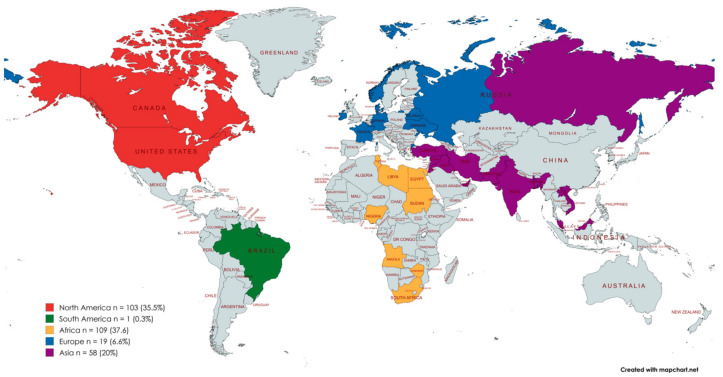
Diversity of participants’ reported countries of origin, displayed by region.

A cohort of 290 medical students were included in the latter analysis. The mean age of the participants was 24.4 ± 3.9 (Me = 24; min = 18; max = 44). The majority of the participants were female (68.2%). A total of 98 (33.8%) respondents attended 9 Canadian universities (CA), 97 (33.4%) attended 2 Libyan universities (LY), and 95 (32.8%) attended 1 Polish university, with admission to programs in English language (PL-Eng). Despite attending medical institutions in only 3 countries, a total of 37 countries were reported by the participants when they were invited to identify their country of origin (Figure 2).

Trained medical students were next divided into subgroups based on their answer to the question, “With which recommendation standards or resuscitation council did the training align?”. From this procedure of guidelines identification, 7 new reference groups were formed, including the American Heart Association (AHA), American Red Cross (ARC), Canadian First Aid Education Guidelines (CFAEG), Canadian Resuscitation & First Aid Guidelines (CRFAG), the European Resuscitation Council (ERC), the Royal Life Saving Society (RLSS), Saint John Ambulance (SJA), and the Resuscitation Council of Southern Africa (RCSA) (Table 2).

Surveys of the participants were divided into 7 groups according to the guidelines on which they were trained, assessed by their answers to open-ended questions, and compared to the untrained group. This study evaluated the participants based on their first and second choice of rescue technique with the clinical scenario questions, “When you encounter an infant who is choking, showing signs of ineffective cough and maintaining consciousness, what should be your first manoeuvre to clear the airway?” (Infant—First Manoeuvre), and “When your first manoeuvre is unsuccessful and the infant is still choking, what should be your second manoeuvre to clear the airway?” (Infant—Second Manoeuvre). The questionaries also required respondents to specify how they would position the infant each time during the chosen rescue manoeuvre (Table 3).

The survey questions, “When you encounter a 3-year-old child who is choking, showing signs of ineffective cough and maintaining consciousness, what should be your first manoeuvre to clear the airway?” (Child—First Manoeuvre), and “When your first manoeuvre is unsuccessful, what should be your second manoeuvre to clear the airway?” (Child—Second Manoeuvre), assessed the medical students’ knowledge of first aid for children using the same approach (Table 4).

Since the responses were open-ended, the terminology used was often not technical, and the answers had to be categorized. Groups were created accordingly, consisting of three mostly recommended rescue manoeuvres (abdominal thrusts, back blows, chest thrusts), with others categorized in one group (other) which comprised assessing the airway, a blind finger sweep of the oropharynx, encouragement of coughing, and changing the position of the infant for more comfort. As a result, both Table 2 and Table 3 were shortened. The original, more precise tables are included in the Supplementary Materials (Supplementary File S3).

**Figure 3:**
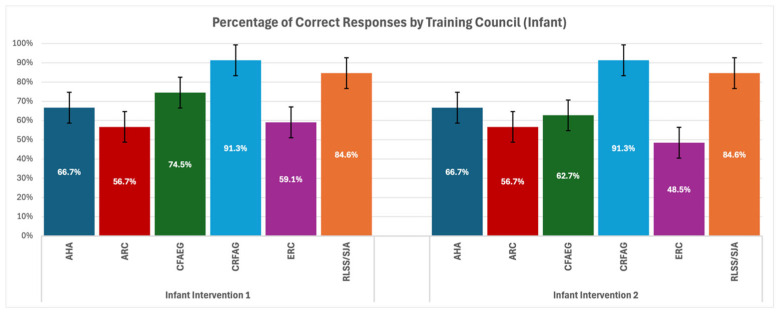
Response adherence by training council for infant interventions.

**Figure 4:**
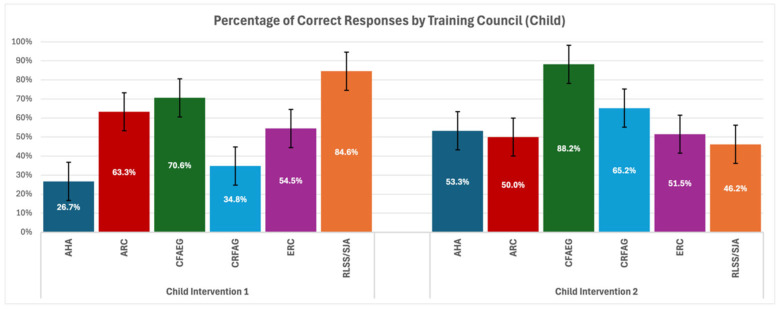
Response adherence by training council for child interventions.

Then, a comparative analysis was carried out to evaluate the adherence of the above-described responses to the specific guidelines and assessed as to whether they were consistent with the training declared by the respondents (Figure 3 and Figure 4).

The data from four different scenarios was analysed through Fisher’s exact test, which is appropriate for small sample sizes and contingency tables with expected frequencies less than 5. The results indicate the following:

- Infant—First Manoeuvre:

Fisher’s exact test resulted in a *p*-value of 0.007659, indicating that there are statistically significant differences among the different training councils in the adherence to the first rescue manoeuvre in a choking infant scenario. This suggests that some guidelines may perform better than others in this clinical situation. However, the post hoc analysis showed no significant differences between the specific pairs of groups after adjusting for multiple comparisons (FDR correction), implying that while there is an overall significant effect, the differences between any individual training council were not significant at the 95% confidence level.

- Infant—Second Manoeuvre:

For the second rescue manoeuvre for helping a choking infant scenario, the *p*-value of 0.001414 suggests a significant effect, indicating differences in adherence to the algorithm for infants between the different training councils. Post hoc testing revealed that there is a statistically significant difference between ERC and CRFAG councils (*p* = 0.00794), showing that guidelines from different councils may lead to notable variations in the application of the algorithm.

- Child—First Manoeuvre:

For the first rescue manoeuvre for a conscious child who is choking, the *p*-value of 0.001192 indicates differences in the adherence rates. Post hoc analyses highlighted significant pairwise differences between the following:

- AHA vs. CFAEG (adjusted *p* = 0.0304);
- AHA vs. RLSS/SJA (adjusted *p* = 0.0304);
- CFAEG vs. CRFAG (adjusted *p* = 0.0304);
- CRFAG vs. RLSS/SJA (adjusted *p* = 0.0304).

These findings suggest that the respondents trained by opposed training councils differ in their choice of first rescue manoeuvre for choking children. In these pairs, CFAEG, RLSS/SJA, CFAEG, and RLSS/SJA, respectively, performed better.

- Child—Second Manoeuvre:

Fisher’s exact test yielded a *p*-value of 0.0001921, indicating a high statistical difference in adherence between the councils. The FDR post hoc analysis revealed significant differences between the following:

- AHA vs. CFAEG (adjusted *p* = 0.034);
- ARC vs. CFAEG (adjusted *p* = 0.00432);
- CFAEG vs. ERC (adjusted *p* = 0.000479);
- CFAEG vs. RLSS/SJA (adjusted *p* = 0.0182).

This indicates that some respondents have a stronger adherence to the algorithm for second rescue manoeuvre for choking children than others, with CFAEG performing notably better in several pairwise comparisons.

**Figure 5:**
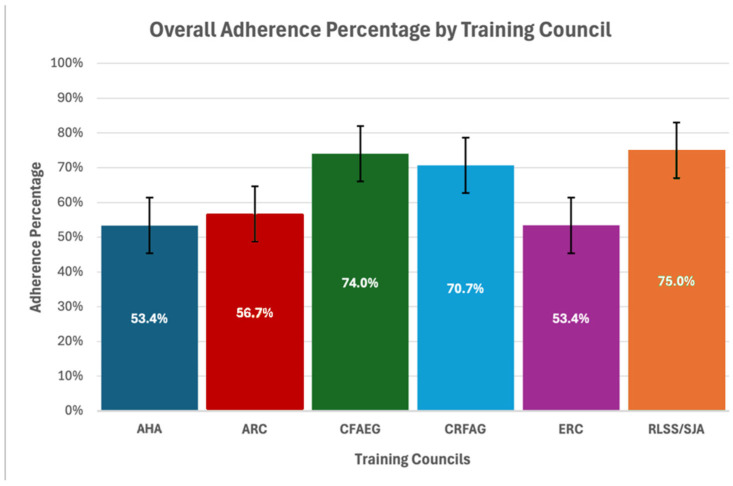
Overall adherence percentage by training council.

Figure 5 was created to better visualize the overall adherence percentage for each training council, combining the results from all four scenarios. 

Additionally, the respondents were also asked, “What steps would you take if an infant or a child who is choking loses consciousness and stops to breathe—explain the sequence of actions that should be followed”, in order to examine their ability to recognize cardiac arrest caused by FBAO and implement CPR (Table 5).

Furthermore, the problems of anti-choking suction devices (ACSDs), medical follow-ups, and blind finger sweeping were explored. Participants were asked the questions, “Is it recommended to utilize anti-choking suction devices in the first aid of a choking child?”, “If the foreign body airway obstruction is relieved, is it necessary to routinely seek urgent medical follow-up from a professional?”, and “Would you attempt a blind finger sweep manoeuvre in this scenario to attempt to clear the obstruction by using your finger to remove not visible object?” (Table 6). 

## 4. Discussion

This study reveals substantial variability in knowledge retention across training councils, with RLSS/SJA consistently outperforming others. However, the small sample size in this group makes these findings less generalizable. CFAEG achieved the highest adherence rates on average in most scenarios. This highlights the efficacy of their training approach, likely attributed to detailed, simulation-based curricula that reinforce long-term retention. In contrast, AHA, while globally prominent, showed relatively poor adherence in Child—First Manoeuvre, indicating potential gaps in their teaching methodologies for paediatric choking scenarios. Similarly, ERC displayed moderate but inconsistent results for Infant—Second Manoeuvre. CRFAG performed exceptionally well in infant scenarios yet had lower success rates with first rescue manoeuvres for child choking. This disparity may reflect differences in emphasis during training or the complexity of certain algorithms.

In clinical scenario questions regarding helping a choking infant and choosing first rescue manoeuvres, we found significant differences among the councils; this suggests that some guidelines may be more effective in training. Although no specific pairwise differences were significant in the post hoc analysis, this still indicates that the chosen guidelines significantly influence performance. For questions regarding the choice of the second rescue manoeuvre, we found significant differences between CRFAG and ERC groups; this implies that some councils might have more effective methods for teaching. It would be beneficial to explore these differences further to refine training protocols globally. Furthermore, clinical scenario questions regarding helping a choking child and choosing first rescue manoeuvres found significant findings that suggest certain councils (such as CFAEG) may provide superior training, which leads to better adherence to choking management procedures. Sharing best practices among councils could help improve global outcomes. In questions where medical students had to choose a second rescue manoeuvre, very significant differences between councils where found, which emphasizes the need for standardized training materials that ensure higher consistency in the application of emergency procedures. CFAEG and CRFAG, in particular, performed well and might serve as a model for improving other councils’ protocols.

In all of the aforementioned clinical scenario questions, an additional assessment of positioning in which chosen rescue manoeuvres would be performed was carried out. This consideration has never been studied before, and no scientific evidence exist; however, it seems plausible that positioning may have some impact on the effectiveness and safety of the rescue manoeuvre, due to the influence of gravity on the foreign body and a more convenient anatomical positioning of the airway. This can potentially occur when a victim is placed in a position with a forward lean, and their head is pointed downwards [33]. Additional aspects of ergonomics exist, e.g., placing the child on the rescuer’s lap or forearm [34,35]. In Infant—First Manoeuvre, a significant portion of the respondents understood this concept. However, in Infant—Second Manoeuvre, some difficulties occurred, most likely caused by doubts regarding the rescue manoeuvres themselves. In Child—First Manoeuvre and Child—Second Manoeuvre, the respondents were more in agreement that the victim should be standing with a forward lean.

The use of Fisher’s exact test was appropriate given the small sample sizes and non-normal distributions. The significant *p*-values suggest strong evidence that different councils do indeed lead to different outcomes in choking scenario adherence. However, the pairwise comparisons (post hoc tests) showed that only certain groups had significant differences, which suggests that while there is a general trend, the differences are not uniform across all comparisons.

In the question about a child who experienced a sudden cardiac arrest after a choking episode, CPR was attempted by 64.1% of the respondents, which is a worrying result. The best performance was achieved by the CRFAG group and RLS/SJA and RCSA, although the latter were significantly limited in the number of participants. The remaining groups achieved a similar result, not distinguishing themselves significantly from each other. A small group noted that they would call for help from professionals; CRFAG and RLS/SJA stood out, with an average response of 65.2% and 79.6%, respectively. The remaining respondents would implement chest thrusts or back blows or did not know what to do in this situation, or their answers were considered incorrect, because they are not recommended in any guidelines. The importance of CPR in unresponsive patients after a choking incident has been established and supported by research repeatedly. Choking followed by severe brain tissue hypoxia causes cardiac arrest with extremely poor prognosis, especially when patients are not immediately resuscitated by witnesses through first aid [27,36].

Additional aspects of first aid in FBAO, such as ACSDs, medical follow-ups, and blind finger sweeping, were explored in order to select the group of respondents with the best level of understanding of these subsidiary issues. The guidelines gathered in this study are consistent in their recommendations regarding the above aspects. Firstly, the utilization of ACSDs gave a lot of doubts to the medical students, which may be due to the fact that many guidelines do not emphasize this topic enough [23,24,25,26,27,28,29,30]. They are not recommended due to lack of evidence, but there is a growing trend of describing and studying them in the scientific literature [22,37,38]. The respondents from CRFAG, RLSS/SJA, and ARC had the highest percentage of correct answers, while the rest were mostly incorrect or unsure. Secondly, the position on medical follow-ups after the application of rescue manoeuvres is also well unified across the guidelines. As FBAO can cause airway trauma and external forces applied during assistance procedures may also result in injuries, it is recommended to consult a physician after such an event. However, not all respondents had this information well assimilated, as less than half of the AHA and ARC groups chose not to seek a medical follow-up, performing worse than the untrained individuals. Thirdly, the knowledge and attitude toward blind finger sweeping the oropharynx was assessed to determine the quality of the guidelines learned, as all training councils do not support it. Again, the greatest awareness of the problem was shown by the respondents trained by CRFAG and RLSS/SJA. The worst performers were the students from the AHA and ERC groups, almost at the level of the untrained students. The RCSA group was willing to use this procedure, but it must be remembered that this was a very small and unrepresentative group.

This study’s findings also highlight the importance of training, as the untrained respondents provided responses that were significantly poorer, nearly double that of the trained respondents. The majority of the untrained respondents answered, “I am not sure”. This seems to be consistent with existing evidence [39,40,41]. The sole existence of medical students that are not trained in basic first aid is very worrying. Three countries partook in the study, and in each, first aid is embedded and taught across the academic curriculum or required as a program prerequisite for entry. However, the volunteers who took part in the study came from different nations and individual backgrounds, meaning that their origins further remain unknown. This prompts a reflection of the educational systems of not only medical students but also of children and youth with regard to teaching and learning first aid with the aim of improving patient survival of FBAO. 

This study is among the first to map medical student knowledge of paediatric FBAO interventions against specific training council guidelines across multiple countries. By identifying clear differences in adherence tied to curricular exposure, the findings provide empirical support for harmonizing guideline content in medical education. Rather than broadly identifying knowledge gaps, this study points to specific areas, such as confusion around back blows versus abdominal thrusts, where targeted educational improvements could improve consistency and safety in paediatric emergency response.

### 4.1. Proposal of Practical Implications

- Focus on Standardization: Given the variability in adherence rates, harmonizing training protocols across international councils could bridge knowledge gaps and improve outcomes globally.
- Enhanced Training Techniques: The superior performance of CFAEG and CRFAG highlights the importance of simulation-based training, which may serve as a model for other organizations.
- Targeted Interventions for Underperforming Regions: Customizing training to the local context, as well as increasing accessibility to high-quality materials, could help underperforming councils improve.
- Periodic Assessments: Longitudinal studies to assess knowledge retention over time and its correlation with real-world competence should be prioritized.

### 4.2. Recommendations for Future Research

There are significant differences in adherence to guideline-recommended manoeuvres, with some councils demonstrating superior outcomes. Future research should focus on follow-up studies analysing the instructional methods employed by these councils and how they differ from others. Research should focus on exploring the reasons that could not be examined in this study, such as a deeper analysis of training methods (e.g., simulation-based or highly structured pedagogical models), cultural differences in training acceptance, and the impact of refresher courses or long-term retention. It may also be valuable to investigate how training delivery (e.g., in person vs. online) impacts adherence to guidelines, as this could be another contributing factor to the observed differences.

## 5. Limitations

This study has several limitations. Firstly, the problem of guideline identification arose during the statistical analysis and assigning respondents to the groups, as some of them could not identify their training council. As a result, a number of the questionnaires had to be excluded. This could be corrected in future studies by focusing their scope to a specific group of individuals who are aware of their training or where their training is known. Secondly, the questionnaires did not examine the year or level of education of the respondents, which could potentially have some effects on their knowledge; however, it is unlikely that this would have any influence on their adherence to guidelines or training councils. On the other hand, further examination of knowledge retention by assessing factors such as the year of medical training could enhance the findings. Additionally, other potential confounding factors were not extensively examined. These include institutional differences across universities, potential differences in education curriculum and level, graduating from another medical speciality in the past, and different course methodologies. Thirdly, this study did not assess encouraging coughing in conscious children with an effective cough, as this action is not an actual rescue manoeuvre and is typically straightforward and universally recommended, being less subject to variation in medical education. Fourthly, the data was gathered from three countries, which gives some collective value to the findings. Nevertheless, not all of it should be applied globally, as the limited geographic and institutional distribution may restrict the results from broader applications. Fifthly, the responses were based on self-assessments and participant recall, which may have been influenced by the social desirability bias or may be inaccurate due to limitations in memory. These factors, along with the exploratory nature of preferred body positioning during rescue manoeuvres and attitudes toward anti-choking suction devices, should prompt cautious interpretation due to the lack of practical validation and differences in the groups’ sample sizes.

## 6. Conclusions

This study identified observable differences in knowledge and adherence to paediatric choking rescue guidelines among medical students trained under different international standards. Certain educational institutions manage to produce better results in terms of adherence to choking intervention guidelines regarding primary and secondary first aid rescue manoeuvres as well as cardiopulmonary resuscitation for children and infants. These descriptive findings may reflect underlying variations in the structure and content of training curricula. Although no multivariate analysis was performed, the results suggest that curricular harmonization might be a useful direction to explore in future studies aiming to improve consistency and preparedness in paediatric emergency response. Additional differences were discovered in the degree of awareness regarding such problems like body positioning, anti-choking suction devices, the blind finger sweep manoeuvre, and the need for medical follow-ups for a child with restored airway patency. Further research is needed to understand the factors that lead to such differences in training program outcomes and how they can be minimized to improve global choking management quality.

## Figures and Tables

**Table 1:** Comparison of algorithm differences in recommended manoeuvres and their sequence for managing foreign body airway obstruction in children older than 1 year old who have preserved consciousness but an ineffective cough.

	Back Blows	Chest Thrusts	Abdominal Thrusts
AHA ^1^	not recommended	not recommended	only
ARC ^2^	first	not recommended	second
CFAEG ^3^	combine any two of the three options		
CRFAG ^4^	not recommended	not recommended	only
ERC ^5^	first	not recommended	second
RCSA ^6^	second	not recommended	first
RLSS ^7^/SJA ^8^	first	not recommended	second

^1^ AHA, American Heart Association; ^2^ ARC, American Red Cross; ^3^ CFAEG, Canadian First Aid Education Guidelines; ^4^ CRFAG, Canadian Resuscitation & First Aid Guidelines; ^5^ ERC, European Resuscitation Council; ^6^ RCSA, Resuscitation Council of Southern Africa; ^7^ RLSS, Royal Life Saving Society; ^8^ SJA, Saint John Ambulance.

**Table 2:** Participant characteristics.

N ^1^ = 290 n ^2^ (%)	Canada	Libya	Polish–English Division	Total
Continent of Origin				
North America	98 (95.1)	0 (0)	5 (4.9)	103 (35.5)
Africa	0 (0)	89 (81.7)	20 (18.3)	109 (37.6)
Asia	0 (0)	6 (10.3)	52 (89.7)	58 (20)
Europe	0 (0)	2 (10.5)	17 (89.5)	19 (6.6)
South America	0 (0)	0 (0)	1 (100.0)	1 (0.3)
Training Council				
AHA ^3^	0 (0)	6 (40.0)	9 (60.0)	15 (4.8)
ARC ^4^	1 (3.3)	29 (96.7)	0 (0)	30 (9.6)
CFAEG ^5^	51 (100)	0 (0)	0 (0)	51 (16.4)
CRFAG ^6^	23 (100)	0 (0)	0 (0)	23 (7.4)
ERC ^7^	0 (0)	4 (6.1)	62 (93.9)	66 (21.2)
RLSS ^8^/SJA ^9^	13 (100)	0 (0)	0 (0)	13 (4.2)
RCSA ^10^	0 (0)	0 (0)	2 (100)	2 (0.6)
Untrained	10 (11.1)	58 (64.4)	22 (24.4)	90 (28.9)

^1^ N, population size; ^2^ n, sample size; ^3^ AHA, American Heart Association; ^4^ ARC, American Red Cross; ^5^ CFAEG, Canadian First Aid Education Guidelines; ^6^ CRFAG, Canadian Resuscitation & First Aid Guidelines; ^7^ ERC, European Resuscitation Council; ^8^ RCSA, Resuscitation Council of Southern Africa; ^9^ RLSS, Royal Life Saving Society; ^10^ SJA, Saint John Ambulance.

**Table 3:** Participant responses to clinical scenario questions by group (infant).

An Infant is Choking, Showing Signs of Ineffective Cough and Maintaining Consciousness. What Would be Your First Manoeuvre to Clear the Airway? (Infant—First Manoeuvre)									
Primary Intervention	AHA ^1^	ARC ^2^	CFAEG ^3^	CRFAG ^4^	ERC ^5^	RCSA ^6^	RLSS ^7^/SJA ^8^	Untrained	Total
n ^9^ (%)	15	30	51	23	66	2	13	90	290
Abdominal thrusts	1 (6.7)	0 (0)	0 (0)	0 (0)	4 (6.1)	0 (0)	0 (0)	7 (7.8)	12 (4.1)
Back blows	10 (66.7)	17 (56.7)	38 (74.5)	21 (91.3)	39 (59.1)	1 (50)	0 (0)	30 (33.3)	166 (57.2)
Chest thrusts	0 (0)	1 (3.3)	7 (13.7)	1 (4.3)	7 (10.6)	1 (50)	11 (84.6)	3 (3.3)	21 (7.2)
Other	4 (26.7)	12 (40)	6 (11.8)	1 (4.3)	16 (24.2)	0 (0)	2 (15.4)	50 (55.6)	91 (31.4)
Positioning during manoeuvre									
Lying flat on the ground	1 (6.7)	2 (6.7)	1 (2)	0 (0)	6 (9.1)	0 (0)	0 (0)	4 (4.4)	14 (4.8)
Lying flat on your lap	0 (0)	2 (6.7)	4 (7.8)	0 (0)	7 (10.6)	0 (0)	0 (0)	18 (20)	31 (10.7)
Lying on your lap with its head downwards	14 (93.3)	26 (86.7)	46 (90.2)	23 (100)	53 (80.3)	2 (100)	13 (100)	68 (75.6)	245 (84.5)
What would be your second manoeuvre to clear the airway if the first manoeuvre was unsuccessful and the patient’s status is unchanged? (Infant—Second Manoeuvre)									
Abdominal thrusts	0 (0)	3 (10)	1 (2)	0 (0)	10 (15.2)	0 (0)	0 (0)	11 (12.2)	25 (8.6)
Back blows	2 (13.3)	7 (23.3)	16 (31.4)	2 (8.7)	9 (13.6)	2 (100)	2 (15.4)	14 (15.6)	54 (18.6)
Chest thrusts	10 (66.7)	17 (56.7)	32 (62.7)	21 (91.3)	32 (48.5)	0 (0)	11 (84.6)	19 (21.1)	142 (49)
Other	3 (20)	3 (10)	2 (3.9)	0 (0)	15 (22.7)	0 (0)	0 (0)	46 (51.1)	69 (23.8)
Positioning during manoeuvre									
Lying flat on the ground	3 (20)	5 (16.7)	0 (0)	0 (0)	7 (10.6)	0 (0)	0 (0)	18 (20)	33 (11.4)
Lying flat on your lap	9 (60)	9 (30)	16 (31.4)	2 (8.7)	31 (47)	0 (0)	2 (15.4)	23 (25.6)	92 (31.7)
Lying on your lap with its head downwards	3 (20)	16 (53.3)	35 (68.6)	21 (91.3)	28 (42.4)	2 (100)	11 (84.6)	49 (54.4)	165 (56.9)

^1^ AHA, American Heart Association; ^2^ ARC, American Red Cross; ^3^ CFAEG, Canadian First Aid Education Guidelines; ^4^ CRFAG, Canadian Resuscitation & First Aid Guidelines; ^5^ ERC, European Resuscitation Council; ^6^ RCSA, Resuscitation Council of Southern Africa; ^7^ RLSS, Royal Life Saving Society; ^8^ SJA—Saint John Ambulance; ^9^ n, sample size.

**Table 4:** Participant responses to clinical scenario questions by group (child).

A 3-Year-Old Child is Choking, Showing Signs of Ineffective Cough and Maintaining Consciousness. What Would be Your First Manoeuvre to Clear the Airway? (Child—First Manoeuvre)									
Primary Intervention	AHA ^1^	ARC ^2^	CFAEG ^3^	CRFAG ^4^	ERC ^5^	RCSA ^6^	RLSS ^7^/SJA ^8^	Untrained	Total
n ^9^ (%)	15	30	51	23	66	2	13	90	290
Abdominal thrusts	4 (26.7)	4 (13.3)	13 (25.5)	8 (34.8)	13 (19.7)	0 (0)	5 (38.5)	21 (23.3)	68 (23.4)
Back blows	8 (53.3)	17 (56.7)	23 (45.1)	13 (56.5)	36 (54.5)	0 (0)	8 (61.5)	25 (27.8)	130 (44.8)
Chest thrusts	0 (0)	0 (0)	0 (0)	0 (0)	1 (1.5)	0 (0)	0 (0)	0 (0)	1 (0.3)
Other	3 (20)	9 (30)	15 (29.4)	2 (8.7)	17 (24.3)	2 (100)	0 (0)	44 (48.9)	91 (31.5)
Positioning during manoeuvre									
	0 (0)	2 (6.7)	1 (2)	0 (0)	6 (9.1)	0 (0)	0 (0)	4 (4.4)	14 (4.8)
Sitting upright	1 (6.7)	1 (3.3)	0 (0)	0 (0)	4 (6.1)	0 (0)	0 (0)	8 (8.9)	14 (4.8)
Sitting with a forward lean	4 (26.7)	4 (13.3)	1 (2)	0 (0)	8 (12.1)	0 (0)	1 (7.7)	16 (17.8)	34 (11.7)
Standing upright	3 (20)	3 (10)	9 (17.6)	6 (26.1)	4 (6.1)	0 (0)	4 (30.8)	16 (17.8)	45 (15.5)
Standing upright with a forward lean	7 (46.7)	22 (73.3)	41 (80.4)	17 (73.9)	50 (75.8)	2 (100)	8 (61.5)	50 (55.6)	197 (67.9)
What would be your second manoeuvre to clear the airway if the first manoeuvre was unsuccessful and the patient’s status is unchanged? (Child—Second Manoeuvre)									
Abdominal thrusts	8 (53.3)	15 (50)	25 (49)	15 (65.3)	34 (51.5)	0 (0)	6 (46.1)	23 (25.5)	126 (43.4)
Back blows	1 (6.7)	4 (13.3)	18 (35.3)	7 (30.4)	9 (13.6)	2 (100)	5 (38.5)	9 (10)	55 (19.0)
Chest thrusts	1 (6.7)	3 (10)	7 (13.7)	1 (4.3)	6 (9.1)	0 (0)	2 (15.4)	3 (3.3)	23 (7.9)
Other	5 (33.3)	8 (26.7)	1 (2)	0 (0)	17 (25.8)	0 (0)	0 (0)	55 (61)	86 (29.7)
Positioning during manoeuvre									
Sitting upright	0 (0)	1 (3.3)	0 (0)	0 (0)	0 (0)	0 (0)	1 (7.7)	7 (7.8)	9 (3.1)
Sitting with a forward lean	0 (0)	5 (16.7)	1 (2)	0 (0)	8 (12.1)	0 (0)	0 (0)	24 (26.7)	38 (13.1)
Standing upright	3 (20)	7 (23.3)	16 (31.4)	9 (39.1)	24 (36.4)	0 (0)	3 (23.1)	16 (17.8)	78 (26.9)
Standing upright with a forward lean	12 (80)	17 (56.7)	34 (66.7)	14 (60.9)	34 (51.5)	2 (100)	9 (69.2)	43 (47.8)	165 (56.9)

^1^ AHA, American Heart Association; ^2^ ARC, American Red Cross; ^3^ CFAEG, Canadian First Aid Education Guidelines; ^4^ CRFAG, Canadian Resuscitation & First Aid Guidelines; ^5^ ERC, European Resuscitation Council; ^6^ RCSA, Resuscitation Council of Southern Africa; ^7^ RLSS, Royal Life Saving Society; ^8^ SJA—Saint John Ambulance; ^9^ n, sample size.

**Table 5:** Participant responses to clinical questions by group (CPR).

Primary Intervention	AHA ^1^	ARC ^2^	CFAEG ^3^	CRFAG ^4^	ERC ^5^	RCSA ^6^	RLSS ^7^/SJA ^8^	Untrained	Total
n ^9^ (%)	15	30	51	23	66	2	13	90	290
What steps would you take if an infant or a child is choking, loses consciousness, and stops breathing									
Request help/EMS ^10^	7 (46.7)	8 (26.7)	20 (39.2)	15 (65.2)	26 (39.4)	0 (0)	10 (79.6)	17 (18.9)	103 (35.5)
CPR ^11^	10 (66.7)	21 (70)	39 (76.5)	23 (100)	46 (69.7)	2 (100)	13 (100)	32 (35.6)	186 (64.1)
Chest thrusts or back blows	1 (6.7)	5 (16.7)	9 (17.6)	0 (0)	10 (15.2)	0 (0)	0 (0)	8 (8.9)	33 (11.4)
Unsure	1 (6.7)	5 (16.7)	3 (5.9)	0 (0)	4 (6.1)	0 (0)	0 (0)	36 (40)	49 (16.9)

^1^ AHA, American Heart Association; ^2^ ARC, American Red Cross; ^3^ CFAEG, Canadian First Aid Education Guidelines; ^4^ CRFAG, Canadian Resuscitation & First Aid Guidelines; ^5^ ERC, European Resuscitation Council; ^6^ RCSA, Resuscitation Council of Southern Africa; ^7^ RLSS, Royal Life Saving Society; ^8^ SJA, Saint John Ambulance; ^9^ n, population; ^10^ EMS, Emergency Medical Services; ^11^ CPR, Cardiopulmonary Resuscitation.

**Table 6:** Participant responses to clinical questions by group (ACSDs, medical follow-ups, and blind finger sweeping).

Primary Intervention	AHA ^1^	ARC ^2^	CFAEG ^3^	CRFAG ^4^	ERC ^5^	RCSA ^6^	RLSS ^7^/SJA ^8^	Untrained	Total
n ^9^ (%)	15	30	51	23	66	2	13	90	290
Is it recommended to utilize anti-choking suction devices in the first aid of a choking child?									
Yes	3 (20)	3 (10)	3 (5.9)	0 (0)	13 (19.7)	2 (100)	0 (0)	24 (26.7)	48 (16.6)
No	2 (13.4)	15 (50)	18 (35.3)	15 (65.2)	13 (19.7)	0 (0)	8 (61.5)	10 (11.1)	81 (27.9)
Unsure	10 (66.7)	12 (40)	30 (58.8)	8 (34.8)	40 (60.6)	0 (0)	5 (38.5)	56 (62.2)	161 (55.5)
If the foreign body airway obstruction is relieved, is it necessary to routinely seek urgent medical follow-up?									
Yes	6 (40)	11 (36.7)	47 (92.2)	19 (82.6)	46 (70.7)	2 (100)	10 (76.9)	55 (61.1)	196 (67.6)
No	6 (40)	8 (26.7)	2 (3.9)	2 (8.7)	11 (16.7)	0 (0)	1 (7.7)	12 (13.3)	42 (14.5)
Unsure	3 (20)	11 (36.7)	2 (3.9)	2 (8.7)	9 (13.6)	0	2 (15.4)	23 (25.6)	52 (17.9)
Would you perform a blind finger sweep to remove an unseen obstruction?									
Yes	3 (20)	1 (3.3)	6 (11.8)	0 (0)	15 (22.7)	2 (100)	0 (0)	15 (16.7)	42 (14.5)
No	7 (46.7)	23 (76.7)	38 (74.5)	22 (95.7)	37 (56.1)	0 (0)	12 (92.3)	39 (43.3)	178 (61.4)
Unsure	5 (33.4)	6 (20)	7 (13.7)	1 (4.3)	14 (21.2)	0 (0)	1 (7.7)	36 (40)	70 (24.1)

^1^ AHA, American Heart Association; ^2^ ARC, American Red Cross; ^3^ CFAEG, Canadian First Aid Education Guidelines; ^4^ CRFAG, Canadian Resuscitation & First Aid Guidelines; ^5^ ERC, European Resuscitation Council; ^6^ RCSA, Resuscitation Council of Southern Africa; ^7^ RLSS, Royal Life Saving Society; ^8^ SJA, Saint John Ambulance; ^9^ n, population.

## Data Availability

The data presented in this study is available on reasonable request from the corresponding author due to restrictions resulting from the policy of the Bioethics Committee.

## Supplementary Materials

The following supporting information can be downloaded at: https://www.mdpi.com/article/10.3390/healthcare13121441/s1, File S1: STROBE checklist; File S2: Online survey questionnaire; File S3: Table S1: Participant responses to clinical scenario questions by group (infant); Table S2: Participant responses to clinical scenario questions by group (child).
