# Supplementary material for: Medical Students’ Knowledge and Adherence to Paediatric Choking Rescue Manoeuvre Guidelines: A Multicentre Study of Medical Education Curricula
Source: Healthcare (Basel). 2025 Jun 16;13(12):1441. doi: 10.3390/healthcare13121441 (PMC12192562; doi:10.3390/healthcare13121441)
Supplement: Supplementary file 1 [file healthcare-13-01441-s001.zip › Supplementary File S1.pdf]

STROBE Statement—checklist of items that should be included in reports of observational studies

	Item No.	Recommendation	Page No.	Relevant text from manuscript
**Title and abstract**	1	(*a*) Indicate the study's design with a commonly used term in the title or the abstract	1	The title includes 'Multicentre Study' and the abstract specifies a 'cross-sectional study'.
		(*b*) Provide in the abstract an informative and balanced summary of what was done and what was found	1	Abstract includes objectives, methods, results, and conclusion, providing a balanced summary.
**Introduction**				
Background/rationale	2	Explain the scientific background and rationale for the investigation being reported	2-3	Introduction outlines the global burden of paediatric choking and lack of training among students.
Objectives	3	State specific objectives, including any prespecified hypotheses	3-4	Objectives stated explicitly at the end of the introduction.
**Methods**				
Study design	4	Present key elements of study design early in the paper	4	Cross-sectional design described in section 2.1.
Setting	5	Describe the setting, locations, and relevant dates, including periods of recruitment, exposure, follow-up, and data collection	4-5	Recruitment settings and dates provided (April–July 2024; Canada, Libya, Poland).
Participants	6	(*a*) *Cohort study*—Give the eligibility criteria, and the sources and methods of selection of participants. Describe methods of follow-up<br>*Case-control study*—Give the eligibility criteria, and the sources and methods of case ascertainment and control selection. Give the rationale for the choice of cases and controls<br>*Cross-sectional study*—Give the eligibility criteria, and the sources and methods of selection of participants	5	Eligibility criteria and participant recruitment from medical faculties are described in section 2.3.
		(*b*) *Cohort study*—For matched studies, give matching criteria and number of exposed and unexposed	N/A	Not applicable to cross-sectional design.

		Case-control study—For matched studies, give matching criteria and the number of controls per case		
Variables	7	Clearly define all outcomes, exposures, predictors, potential confounders, and effect modifiers. Give diagnostic criteria, if applicable	5	Outcomes and variables defined in section 2.2 (knowledge and adherence scores).
Data sources/ measurement	8*	For each variable of interest, give sources of data and details of methods of assessment (measurement). Describe comparability of assessment methods if there is more than one group	*5-6*	*Structured questionnaire with validation process and reliability testing described.*
Bias	9	Describe any efforts to address potential sources of bias	6,13	Bias minimized via questionnaire validation, training of data collectors, and pilot testing.
Study size	10	Explain how the study size was arrived at	5	Sample size based on convenience sampling; no formal calculation.
Quantitative variables	11	Explain how quantitative variables were handled in the analyses. If applicable, describe which groupings were chosen and why	6	Quantitative variables analyzed as frequencies, proportions, and comparison of groups.
Statistical methods	12	(*a*) Describe all statistical methods, including those used to control for confounding	6	Chi-square and Fisher's exact test used for group comparisons.
		(*b*) Describe any methods used to examine subgroups and interactions	9-10	Post-hoc tests between countries and academic years described in section 3.
		(*c*) Explain how missing data were addressed	6	Missing data excluded from analysis; noted in methods.
		(*d*) *Cohort study*—If applicable, explain how loss to follow-up was addressed<br>*Case-control study*—If applicable, explain how matching of cases and controls was addressed<br>*Cross-sectional study*—If applicable, describe analytical methods taking account of sampling strategy	N/A	No loss to follow-up due to cross-sectional design.
		(*e*) Describe any sensitivity analyses	N/A	No sensitivity analysis

				conducted.
**Results**				
Participants	13*	(a) Report numbers of individuals at each stage of study—eg numbers potentially eligible, examined for eligibility, confirmed eligible, included in the study, completing follow-up, and analysed	6-7	Initial n=324, final n=290 after exclusion of incomplete responses.
		(b) Give reasons for non-participation at each stage	6	Reasons for exclusion (e.g. incomplete or inconsistent data) are stated.
		(c) Consider use of a flow diagram	7	Participant flow chart presented in Figure 1.
Descriptive data	14*	(a) Give characteristics of study participants (eg demographic, clinical, social) and information on exposures and potential confounders	7-8	Participant characteristics in Table 2.
		(b) Indicate number of participants with missing data for each variable of interest	6	Missing data for excluded participants described.
		(c) *Cohort study*—Summarise follow-up time (eg, average and total amount)		
Outcome data	15*	*Cohort study*—Report numbers of outcome events or summary measures over time		
		Case-control study—Report numbers in each exposure category, or summary measures of exposure		
		Cross-sectional study—Report numbers of outcome events or summary measures	*8-11*	Main results in Tables 3–6, with narrative summary in Results.
Main results	16	(*a*) Give unadjusted estimates and, if applicable, confounder-adjusted estimates and their precision (eg, 95% confidence interval). Make clear which confounders were adjusted for and why they were included	8-11	Unadjusted comparisons with p-values; no confounder adjustment due to study design.
		(*b*) Report category boundaries when continuous variables were categorized	N/A	No categorized continuous variables.
		(*c*) If relevant, consider translating estimates of relative risk into absolute risk for a meaningful time period		
Other analyses	17	Report other analyses done—eg analyses of subgroups and interactions, and sensitivity analyses	9-10	Subgroup comparisons by country and training exposure in Results.
**Discussion**				

Key results	18	Summarise key results with reference to study objectives	11-12	Summary of main findings in section 4.1 Discussion.
Limitations	19	Discuss limitations of the study, taking into account sources of potential bias or imprecision. Discuss both direction and magnitude of any potential bias	13	Study limitations (sampling method, generalizability, validation issues) discussed in section 5.
Interpretation	20	Give a cautious overall interpretation of results considering objectives, limitations, multiplicity of analyses, results from similar studies, and other relevant evidence	13-14	Interpretation with references to prior studies and recommendations for training programs.
Generalisability	21	Discuss the generalisability (external validity) of the study results	13	Generalizability to other student populations discussed under Limitations.
**Other information**				
Funding	22	Give the source of funding and the role of the funders for the present study and, if applicable, for the original study on which the present article is based	15	Funding section confirms no financial support.

*Give information separately for cases and controls in case-control studies and, if applicable, for exposed and unexposed groups in cohort and cross-sectional studies.

Note: An Explanation and Elaboration article discusses each checklist item and gives methodological background and published examples of transparent reporting. The STROBE checklist is best used in conjunction with this article (freely available on the Web sites of PLoS Medicine at http://www.plosmedicine.org/, Annals of Internal Medicine at http://www.annals.org/, and Epidemiology at http://www.epidem.com/). Information on the STROBE Initiative is available at www.strobe-statement.org.
