# Supplementary material for: Medical Students’ Knowledge and Adherence to Paediatric Choking Rescue Manoeuvre Guidelines: A Multicentre Study of Medical Education Curricula
Source: Healthcare (Basel). 2025 Jun 16;13(12):1441. doi: 10.3390/healthcare13121441 (PMC12192562; doi:10.3390/healthcare13121441)
Supplement: Supplementary file 1 [file healthcare-13-01441-s001.zip › Supplementary File S2.pdf]

First Aid Management of Pediatric Foreign Body Airway Obstruction

Dear participant,
We are conducting research to help answer questions about first aid management of pediatric foreign body airway obstruction.

This questionnaire should take approximately 5–10 minutes to finish, your valuable input is essential to our research. Participation in the survey is voluntary and all responses will remain anonymous. Kindly answer based on your own knowledge without seeking external sources, ensuring that the results are genuine and usable. There is no right or wrong answer, all answers are valuable.

By submitting the completed questionnaire/survey, you are consenting to participate in this research.

Thank you for taking the time to contribute to our work,

Primary Investigators:
Jakub R. Bieliński, PhD Candidate (Medical University of Lodz), jakub.bielinski@stud.umed.lodz.pl
Filip Jaśkiewicz, PhD (Medical University of Lodz), filip.jaskiewicz@umed.lodz.pl

1. What is your gender identity?
    - Woman
    - Man
    - Non-binary
    - Prefer not to say
2. How old are you?
3. Which university are you currently studying at or affiliated with?
4. Have you received training on how to manage airway obstruction caused by foreign objects in children?
    - Yes
    - No
5. Which certifying agency did you take this training through (or which resuscitation council/recommendation standards did it align with)?
6. Have you ever had hands-on/real-life experience providing first aid for a child with an obstructed airway due to a foreign object?
    - If the answer is “yes”, please provide a brief description of your experience. For example, specify the child's age, assessed severity of airway blockage, explain the actions taken, and indicate if the foreign body was successfully removed.
7. When you encounter an infant who is choking, showing signs of weak, ineffective cough and maintaining consciousness, what should be your **first** maneuver to clear the airway?
8. In what position should the infant be placed while performing this first life-saving technique?
    - lying flat on your lap
    - lying on your lap with its head downwards
    - lying flat on the ground

9. When your first maneuver is unsuccessful and the infant is still choking, showing signs of weak, ineffective cough and maintaining consciousness, what should be your **second** maneuver to clear the airway?
10. In what position should the infant be placed while performing this second life-saving technique?
    - lying flat on your lap
    - lying on the your lap with its head downwards
    - lying flat on the ground
11. What steps would you take if an infant or a child is choking, loses consciousness, and stops breathing - briefly explain the sequence of actions that should be followed.
12. When you encounter an 3-year-old child who is choking, showing signs of ineffective cough and maintaining consciousness, what should be your **first** maneuver to clear the airway?
13. In what position should the child be placed while performing this first life-saving technique?
    - standing upright
    - standing with a forward lean
    - sitting upright
    - sitting with a forward lean
14. When your first maneuver is unsuccessful and the 3-year-old child is still choking, showing signs of weak, ineffective cough and maintaining consciousness, what should be your **second** maneuver to clear the airway?
15. In what position should the child be placed while performing this second life-saving technique?
    - standing upright
    - standing with a forward lean
    - sitting upright
    - sitting with a forward lean
16. Would you attempt a blind finger sweep maneuver in this scenario to attempt to clear the obstruction by using your finger to remove an object that is not visible?
    - Yes
    - No
    - I am not sure
17. If the foreign body airway obstruction is relieved, is it necessary to routinely seek urgent medical follow-up from a professional?
    - Yes
    - No
    - I am not sure
18. Is it recommended to utilize anti-choking suction devices in the first aid of a choking child?
    - Yes
    - No
    - I am not sure
