# Supplementary material for: Medical Students’ Knowledge and Adherence to Paediatric Choking Rescue Manoeuvre Guidelines: A Multicentre Study of Medical Education Curricula
Source: Healthcare (Basel). 2025 Jun 16;13(12):1441. doi: 10.3390/healthcare13121441 (PMC12192562; doi:10.3390/healthcare13121441)
Supplement: Supplementary file 1 [file healthcare-13-01441-s001.zip › Supplementary File S3.pdf]

Table S1. Participant Responses to Clinical Scenario Questions by Group (Infant)

An infant is choking, showing signs of ineffective cough and maintaining consciousness. What would be your first manoeuvre to clear the airway? (Infant – First Manoeuvre)

Primary Intervention	AHA[1]	ARC[2]	CFAEG[3]	CRFAG[4]	ERC[5]	RCSA[6]	RLSS[7]/SJA[8]	Untrained	Total
n[9] (%)	15	30	51	23	66	2	13	90	290
Abdominal thrusts	1 (6.7)	0 (0)	0 (0)	0 (0)	4 (6.1)	0 (0)	0 (0)	7 (7.8)	12 (4.1)
Airway assessment	1 (6.7)	7 (23.3)	1 (2)	0 (0)	5 (7.6)	0 (0)	0 (0)	10 (11.1)	24 (8.3)
Back blows	10 (66.7)	17 (56.7)	38 (74.5)	21 (91.3)	39 (59.1)	1 (50)	0 (0)	30 (33.3)	166 (57.2)
Blind finger sweep	0 (0)	0 (0)	0 (0)	1 (4.3)	3 (4.5)	0 (0)	2 (15.4)	9 (10)	15 (5.2)
Chest thrusts	0 (0)	1 (3.3)	7 (13.7)	1 (4.3)	7 (10.6)	1 (50)	11 (84.6)	3 (3.3)	21 (7.2)
Encourage coughing	0 (0)	1 (3.3)	5 (9.8)	0 (0)	0 (0)	0 (0)	0 (0)	1 (1.1)	7 (2.4)
Position infant	1 (6.7)	2 (6.7)	0 (0)	0 (0)	3 (4.5)	0 (0)	0 (0)	5 (5.6)	11 (3.8)
Other (< 10 responses)	1 (6.7)	2 (6.7)	0 (0)	0 (0)	3 (4.5)	0 (0)	0 (0)	3 (3.3)	9 (3.1)
Unsure	1 (6.7)	0 (0)	0 (0)	0 (0)	2 (3)	0 (0)	0 (0)	22 (24.4)	25 (8.6)
	Positioning during manoeuvre								
Lying flat on the ground	1 (6.7)	2 (6.7)	1 (2)	0 (0)	6 (9.1)	0 (0)	0 (0)	4 (4.4)	14 (4.8)
Lying flat on your lap	0 (0)	2 (6.7)	4 (7.8)	0 (0)	7 (10.6)	0 (0)	0 (0)	18 (20)	31 (10.7)
Lying on your lap with its head downwards	14 (93.3)	26 (86.7)	46 (90.2)	23 (100)	53 (80.3)	2 (100)	13 (100)	68 (75.6)	245 (84.5)
What would be your second manoeuvre to clear the airway if the first manoeuvre was unsuccessful and the patient's status is unchanged? (Infant – Second Manoeuvre)									
Abdominal thrusts	0 (0)	3 (10)	1 (2)	0 (0)	10 (15.2)	0 (0)	0 (0)	11 (12.2)	25 (8.6)
Back blows	2 (13.3)	7 (23.3)	16 (31.4)	2 (8.7)	9 (13.6)	2 (100)	2 (15.4)	14 (15.6)	54 (18.6)

Chest thrusts	10 (66.7)	17 (56.7)	32 (62.7)	21 (91.3)	32 (48.5)	0 (0)	11 (84.6)	19 (21.1)	142 (49)
Other (< 10 responses)	1 (6.7)	1 (3.3)	1 (2)	0 (0)	7 (10.6)	0 (0)	0 (0)	9 (10)	19 (6.6)
Unsure	2 (13.3)	2 (6.7)	1 (2)	0 (0)	8 (12.1)	0 (0)	0 (0)	37 (41.1)	50 (17.2)
	Positioning during manoeuvre								
Lying flat on the ground	3 (20)	5 (16.7)	0 (0)	0 (0)	7 (10.6)	0 (0)	0 (0)	18 (20)	33 (11.4)
Lying flat on your lap	9 (60)	9 (30)	16 (31.4)	2 (8.7)	31 (47)	0 (0)	2 (15.4)	23 (25.6)	92 (31.7)
Lying on your lap with its head downwards	3 (20)	16 (53.3)	35 (68.6)	21 (91.3)	28 (42.4)	2 (100)	11 (84.6)	49 (54.4)	165 (56.9)

[1] AHA, American Heart Association; [2] ARC, American Red Cross; [3] CFAEG, Canadian First Aid Education Guidelines; [4] CRFAG, Canadian Resuscitation & First Aid Guidelines; [5] ERC, European Resuscitation Council, [6] RCSA, Resuscitation Council of Southern Africa, [7] RLSS, Royal Life Saving Society; [8] SJA - Saint John Ambulance; [9] n, sample size

Table S2. Participant Responses to Clinical Scenario Questions by Group (Child)

A 3-year-old child is choking, showing signs of ineffective cough and maintaining consciousness. What would be your first manoeuvre to clear the airway? (Child – First Manoeuvre)

Primary Intervention	AHA [1]	ARC [2]	CFAEG [3]	CRFAG [4]	ERC [5]	RCSA [6]	RLSS [7]/SJA [8]	Untrained	Total
n [9] (%)	15	30	51	23	66	2	13	90	290
Abdominal thrusts	(26.7)	4 (13.3)	13 (25.5)	8 (34.8)	13 (19.7)	0 (0)	5 (38.5)	21 (23.3)	68 (23.4)
Back blows	8 (53.3)	17 (56.7)	23 (45.1)	13 (56.5)	36 (54.5)	0 (0)	8 (61.5)	25 (27.8)	130 (44.8)
Blind finger sweep	1 (6.7)	1 (3.3)	1 (2)	0 (0)	5 (7.6)	2 (100)	0 (0)	6 (6.7)	16 (5.5)
Chest thrusts	0 (0)	0 (0)	0 (0)	0 (0)	1 (1.5)	0 (0)	0 (0)	0 (0)	1 (0.3)
Encourage coughing	0 (0)	1 (3.3)	13 (25.5)	2 (8.7)	4 (6.1)	0 (0)	0 (0)	3 (3.3)	23 (57.9)
Unsure	1 (6.7)	3 (10)	0 (0)	0 (0)	4 (6.1)	0 (0)	0 (0)	29 (32.2)	15 (5.2)

Other (< 10 responses)	1 (6.7)	4 (13.3)	1 (2)	0 (0)	3 (4.5)	0 (0)	0 (0)	6 (6.7)	37 (12.8)
Positioning during manoeuvre									
Sitting upright	1 (6.7)	1 (3.3)	0 (0)	0 (0)	4 (6.1)	0 (0)	0 (0)	8 (8.9)	14 (4.8)
Sitting with a forward lean	4 (26.7)	4 (13.3)	1 (2)	0 (0)	8 (12.1)	0 (0)	1 (7.7)	16 (17.8)	34 (11.7)
Standing upright	3 (20)	3 (10)	9 (17.6)	6 (26.1)	4 (6.1)	0 (0)	4 (30.8)	16 (17.8)	45 (15.5)
Standing upright with a forward lean	7 (46.7)	22 (73.3)	41 (80.4)	17 (73.9)	50 (75.8)	2 (100)	8 (61.5)	50 (55.6)	197 (67.9)
What would be your second manoeuvre to clear the airway if the first manoeuvre was unsuccessful and the patient's status is unchanged? (Child – Second Manoeuvre)									
Abdominal thrusts	8 (53.3)	15 (50)	25 (49)	15 (65.2)	34 (51.5)	0 (0)	6 (46.2)	23 (25.6)	126 (43.4)
Back blows	1 (6.7)	4 (13.3)	18 (35.3)	7 (30.4)	9 (13.6)	2 (100)	5 (38.5)	9 (10)	55 (19.0)
Chest thrusts	1 (6.7)	3 (10)	7 (13.7)	1 (4.3)	6 (9.1)	0 (0)	2 (15.4)	3 (3.3)	23 (7.9)
Other (< 10 responses)	3 (20)	4 (13.3)	1 (2)	0 (0)	6 (9.1)	0 (0)	0 (0)	11 (12.2)	25 (8.6)
Unsure	2 13.3)	4 (13.3)	0 (0)	0 (0)	11 (16.7)	0 (0)	0 (0)	44 (48.9)	61 (21)
Positioning during manoeuvre									
Sitting upright	0 (0)	1 (3.3)	0 (0)	0 (0)	0 (0)	0 (0)	1 (7.7)	7 (7.8)	9 (3.1)
Sitting with a forward lean	0 (0)	5 (16.7)	1 (2)	0 (0)	8 (12.1)	0 (0)	0 (0)	24 (26.7)	38 (13.1)
Standing upright	3 (20)	7 (23.3)	16 (31.4)	9 (39.1)	24 (36.4)	0 (0)	3 (23.1)	16 (17.8)	78 (26.9)
Standing upright with a forward lean	12 (80)	17 (56.7)	34 (66.7)	14 (60.9)	34 (51.5)	2 (100)	9 (69.2)	43 (47.8)	165 (56.9)

[1] AHA, American Heart Association; [2] ARC, American Red Cross; [3] CFAEG, Canadian First Aid Education Guidelines; [4] CRFAG, Canadian Resuscitation & First Aid Guidelines; [5] ERC, European Resuscitation Council, [6] RCSA, Resuscitation Council of Southern Africa, [7] RLSS, Royal Life Saving Society; [8] SJA - Saint John Ambulance; [9] n, sample size;
